# Prenatal mental health data and birth outcomes in the pregnancy during the COVID-19 Pandemic dataset

**DOI:** 10.1016/j.dib.2023.109366

**Published:** 2023-07-02

**Authors:** Catherine Lebel, Lianne Tomfohr-Madsen, Gerald Giesbrecht, Beatrice Pui Yee Lai, Mercedes Bagshawe, Makayla Freeman, Mary Kate Hapin, Anna MacKinnon, Palak Patel, Melinda van Sloten, Marcel van de Wouw

**Affiliations:** a Department of Radiology, University of Calgary, 2500 University Dr NW, Calgary, AB, Canada; b Department of Education and Counselling Psychology, University of British Columbia, 2125 Main Mall Scarfe Office Block 2528Vancouver, BC V6T 1Z4, Canada; c Department of Pediatrics, University of Calgary, 2500 University Dr NW, Calgary, AB, Canada; d Département de psychiatrie et d'addictologie, Université de Montréal, CHU Sainte Justine Research Centre, 3175 Chemin de la Côte Sainte Catherine, Montréal, QC H3S 2G4, Canada

**Keywords:** Depression, Anxiety, Birthweight, Gestation, Stress

## Abstract

The COVID-19 pandemic was a substantial stressor, especially for pregnant individuals. We aimed to understand the impact of COVID-19-related stresses on pregnant individuals and their infants and collected survey-based data across Canada as part of the Pregnancy during the COVID-19 Pandemic (PdP) project. The dataset described here provides baseline prenatal data and basic birth outcomes from PdP participants. This data includes information from pregnant individuals as well as their infants.

At enrolment and time of completion of the baseline survey, participants were pregnant, ≥17 years of age, ≤35 weeks of gestation, living in Canada, and able to read and write in English or French. Baseline data were collected between April 2020-April 2021. Infant data were collected between May 2020-December 2021. All data were collected via self-report using online questionnaires in REDCAP. Questionnaires were available in both English and French. Data were checked for completeness and plausibility, and duplicates were removed.

The dataset described here includes age, education, and household income of the pregnant individuals reported at the baseline/enrollment survey. Raw scores are provided for the Edinburgh Postnatal Depression Scale (EPDS) and the PROMIS Anxiety scale. Ratings are also given for three variables describing fear of the COVID-19 virus.

Birth outcomes are provided for infants, including gestational age at birth, birthweight, length, mode of delivery, and whether the infant spent time in the neonatal intensive care unit (NICU). Delivery date is reported as month and year.

These data will be beneficial for anyone interested in researching stress during pregnancy or birth outcomes in the context of the COVID-19 pandemic. They will also be useful to researchers interested in examining more general effects of prenatal distress on birth outcomes in children. Data could also be compared to other datasets from the COVID-19 pandemic to establish generalizability, or to pre-pandemic datasets to determine the extent of changes during the COVID-19 pandemic.

**Specifications Table**

**Table:** 

Subject	Psychiatry and Mental Health
Specific subject area	Prenatal mental health and infant birth outcomes
Type of data	Table
How the data were acquired	Prenatal data were acquired as part of the national Pregnancy during the COVID-19 Pandemic cohort study using online questionnaires hosted in REDCap. Demographic data were obtained using basic demographic questions, and mental health was measured with validated self-report questionnaires: the Edinburgh Postnatal Depression Scale [1], and the PROMIS Anxiety scale [2]. Birth and infant data were acquired via parent-report completed in REDCap. Surveys were available in either English or French.
Data format	Raw Filtered
Description of data collection	Participants were recruited via advertisements distributed through various pregnancy organizations and care providers, social media, and via paid ads on Facebook and Instagram, between April 5, 2020-April 9, 2021 [3]. Participants were eligible if they were ≥17 years, pregnant, living in Canada, ≤35 weeks gestation, and able to read and write in English or French. Data is available for 10 772 participants. Those who did not consent, provided insufficient data, provided invalid e-mail addresses, or provided implausible data were excluded from the dataset.
Data source location	*Country:* Canada
Data accessibility	Repository name: Open Science Framework (OSF) Data identification number: Direct URL to data: https://osf.io/ha5dp/
Related research article	Giesbrecht, G.F., Rojas, L., Patel, S., Kuret, V., MacKinnon, A.L., Tomfohr-Madsen, L., Lebel, C., 2022. Fear of COVID-19, mental health, and pregnancy outcomes in the pregnancy during the COVID-19 pandemic study: Fear of COVID-19 and pregnancy outcomes. J Affect Disord 299: 483 [4].

## Value of the Data

• These data provide information on mental health (depression, anxiety, fear) of pregnant individuals during the COVID-19 pandemic, as well as birth outcomes for their infants. The pandemic was a substantial stressor, especially for pregnant individuals and these data help understand that stress and its potential impacts on babies.
• These data will be beneficial for anyone interested in researching stress during pregnancy or birth outcomes in the context of the COVID-19 pandemic and/or the effects of prenatal distress on birth outcomes in children.
• These data can be analyzed on their own or in conjunction with other pregnancy and birth data collected during the pandemic, either in Canada or elsewhere in the world. They could also be compared to similar datasets from prior to or after the COVID-19 pandemic to better understand the specific context of the pandemic.
• The Pregnancy during the COVID-19 Pandemic (PdP) cohort study is ongoing, and we plan to release further data on pregnancy and child development as it is collected and cleaned.

## 1 Objective

Prenatal psychological distress (e.g., depression, anxiety, and stress symptoms) is common during pregnancy, [5,6] and can have long-lasting consequences for pregnant individuals, babies, and families. For example, prenatal distress is associated with a higher risk of postpartum mental health problems, as well as shorter gestation length, lower birthweight, and higher need for surgical birth [7,8]. For infants and children, exposure to prenatal depression or anxiety increases the risk of developmental problems and later mental health challenges, [9], [10], [11] and is associated with brain and behavior changes. [12], [13], [14]

The Pregnancy during the COVID-19 Pandemic (PdP) cohort study was launched in early April 2020 to understand how pregnant individuals were affected by the COVID-19 pandemic and the associated mitigation measures. It aimed to measure mental health, physical health, and life changes among pregnant individuals during the pandemic, and later infant outcomes.

## 2 Data Description

The current Pregnancy during the Pandemic (PdP) dataset on OSF includes two files with the same information in different formats (one SPSS .sav file and one .csv file) as well as a data dictionary. Within the two data files, participants are listed in rows by ID numbers, and each variable is provided in a separate column. The data files contain information from pregnant individuals and basic birth outcomes on their infants.

Prenatal information includes age (in years) at study intake, 2019 household income in Canadian dollars, and education. The language in which the survey was completed (English or French) is also noted. Raw scores are provided for the Edinburgh Postnatal Depression Scale (EPDS) and the PROMIS Anxiety scale. Ratings are given for three different variables describing the fear that COVID-19 would threaten their own lives (threaten_life), threaten their unborn baby's life (threaten_baby_danger), or harm their baby (threaten_baby_harm).

Infant variables include gestational age at birth (in weeks), birthweight (in grams), length (in centimeters), a dichotomous variable (yes/no) for whether the infant spent time in the neonatal intensive care unit (NICU), and the mode of delivery (vaginal or Caesarian). Delivery date is reported as month and year.

## 3 Experimental Design, Materials and Methods

The Pregnancy during the COVID-19 Pandemic (PdP) study is an ongoing study focused on prenatal experiences during the pandemic and subsequent outcomes for parents and children [15,16]. The data currently available on OSF represent measures from the intake (baseline) survey at study enrolment and the birth outcomes reported at the first post-delivery survey. Data from additional prenatal timepoints is currently being cleaned and will be released when available. Similarly, additional infant/child outcomes will be released after collection and cleaning is complete.

*Participants.* At enrolment, participants in the PdP study were pregnant, ≥17 years of age, ≤35 weeks of gestation, living in Canada, and able to read and write in English or French. There were no additional exclusion criteria. The requirement of ≤35 weeks of gestation at study enrollment was intended to allow for multiple data points during a participant's pregnancy, as follow-up surveys were conducted monthly starting 4 weeks after enrolment. Based on their provided due dates, some participants were determined to be >35 weeks of gestation at initial enrollment. Because these participants provided otherwise legitimate data, we retained these datapoints for potential analyses, as relevant.

Participant recruitment began on April 5, 2020 (English survey) and May 1, 2020 (French survey); recruitment ended April 9, 2021 (both surveys). Participants were recruited through social media (Facebook, Instagram, Twitter), through our PdP website, via expectant parents’ groups, prenatal care clinics, and word of mouth. We used paid advertisements in French and English on Facebook and Instagram, with a portion of advertisements targeted to geographic regions and/or sociodemographic groups with less representation in the cohort (e.g., rural communities). Surveys were available in both French and English. For standardized measures, validated French translations were used. For demographic and fear of COVID-19 questions, an official translation service was used to translate, back-translate, and then verify each question. Participants were not provided any reimbursement for the baseline survey; those who completed the delivery survey were entered into a draw for a $500 gift card.

*Prenatal information.* Depression symptoms were measured using the Edinburgh Postnatal Depression Scale (EPDS), which is a valid and reliable measurement of depression symptoms both prenatally and postpartum (sensitivity 0.87, specificity 0.92) [1,17]. The EPDS includes 10 questions each scored on a 4-point scale (0–3). Possible scores range from 0 to 30. Higher scores represent worse depression symptoms.

Anxiety symptoms were measured using the Patient-Reported Outcomes Measurement Information System (PROMIS) [2,18]. The PROMIS Anxiety Adult Short Form includes 7 questions each scored from 1 to 5; possible total scores range from 7 to 35. Higher scores indicate more severe anxiety symptoms.

Three questions specific to fears of COVID-19 were asked. Answers were given on a sliding scale from 0 (“not at all”) to 100 (“very much so”), with a middle anchor at 50 of “somewhat”.

1. How much do (did) you think your life is (was) in danger during the COVID-19 pandemic?
2. How much do (did) you think your unborn baby's life is (was) in danger during the COVID-19 pandemic?
3. How much are (were) you worried that exposure to the COVID-19 virus will (would) harm your unborn baby?

Education was self-reported by category: less than high school diploma, high school diploma, college/trade school, undergraduate degree, masters degree, doctoral degree. According to the 2021 Canadian census, the following percentages of “Women+” (with “Women+” defined as women and some non-binary individuals) aged 25–34 years (the most relevant comparison group) fell into each of these categories: 5.8% less than high school, 17.8% high school diploma, 35.6% college/trade school, 31.3% undergraduate degree, 9.9% Master's degree, 1.9% doctoral [19]. Income was reported as the total household income from all sources in 2019, before taxes and deductions. It was reported on the following scale: <$20,000; $20,000-$39,999; $40,000-$69,999; $70,000-$99,999; $100,000-$124,999; $125,000-$149,999; $150,000-$174,999; $175,000-$199,999; ≥$200,000. Participants provided their birth year and month, which were used to calculate age. For reference, the median after-tax income of Canadian households was $62,900 in 2019 and 10.1% of households lived below Canada's official poverty line (the poverty line varies by province and population density; in 2019, it ranged from $37,397-$48,677) [20].

*Infant information.* Delivery and birth information were collected by parent report following delivery (typically within 2 months). Parents were asked for the date of their infant's birth, which was converted to month and year to preserve anonymity. They were also asked how the infant was delivered (vaginally or Caesarian section), whether the infant spent time in the neonatal intensive care unit (NICU), and to report birth weight, length, and gestational age. Birth length and weight were reported in either metric (centimetres, grams) or imperial (inches, pounds/ounces) units; imperial units were converted to metric for reporting. Gestational age at birth was verified against due dates previously provided by individuals during the intake survey.

*Data checking.* All participant data was screened prior to analysis. Table 1 provides an overview of the data cleaning steps. Cases deemed to be ineligible, providing insufficient information, duplicate entries, or with nonsense information that could not be verified were removed.

**Table 1:** Data checking and verification procedures.

Data checking step	
Screen for eligibility	Participants were screened for eligibility (≥17 years of age, ≤35 weeks of gestation, living in Canada, and able to read and write in English or French). Ineligible participants were removed from the dataset.
Check for insufficient data	Participant records with insufficient data were removed from the dataset. Insufficient data was defined as cases that did not include household income, as that was the last question in the section of essential demographic information.
Check for nonsense responses	Participants with nonsense responses (e.g., one participant recorded ‘meow’ in every text field) were removed. Participants with impossible due dates (e.g., in the past, or >9 months in the future) were contacted for clarification. If due dates could not be corrected, participants were removed from analysis. Participants with other implausible values (e.g., physical activity >16 h/day, very high or very low height or weight, etc.) were also contacted for clarification. If these responses could not be verified, but the other data was plausible, these values were set to missing and participants were retained in the dataset.
Verify email addresses	Records with email addresses that could not be verified (e.g., bounce-back when contacted for follow-up) were removed from the dataset.
Identify duplicate entries	Email address, phone number, postal code, maternal date of birth, and due date were used to identify potential duplicate entries. In the case of duplicate entries, the record with the more complete data was kept.
Check for consistency of responses	The due date, delivery date, and reported gestational age at birth were checked for consistency. Participants were contacted for clarification if they met any of the following criteria: 1. Delivery date occurred more than 3 weeks after the expected due date (i.e., gestational age at birth was > 43 weeks) 2. There were discrepancies in delivery dates across follow-ups and postpartum surveys 3. Delivery date occurred before the expected due date and the reported gestational age at birth did not match calculated gestational age at birth Variables that could not be verified were set to missing.
Other checks	We looked at the time taken to complete the survey, to flag potential bot responses. All participants took >9 min to complete the surveys, and many provided sensible answers to the open-answer questions, suggesting that these were legitimate responses. We looked at cases for patterns of consistency or inconsistency and did not identify any concerning patterns across items that suggested fake answers.

## Ethics Statement

All participants in this study provided informed consent. Research was approved by the University of Calgary conjoint health research ethics board (CHREB; REB20-0500) and conduced in accordance with the Declaration of Helsinki.

## CRediT authorship contribution statement

**Catherine Lebel:** Conceptualization, Methodology, Writing – original draft, Data curation, Supervision. **Lianne Tomfohr-Madsen:** Conceptualization, Methodology, Writing – review & editing, Data curation, Supervision. **Gerald Giesbrecht:** Conceptualization, Methodology, Writing – review & editing, Data curation, Supervision. **Beatrice Pui Yee Lai:** Data curation, Writing – review & editing. **Mercedes Bagshawe:** Methodology, Data curation, Writing – review & editing. **Makayla Freeman:** Methodology, Data curation, Writing – review & editing. **Mary Kate Hapin:** Methodology, Data curation, Writing – review & editing. **Anna MacKinnon:** Methodology, Data curation, Writing – review & editing. **Palak Patel:** Methodology, Data curation, Writing – review & editing. **Melinda van Sloten:** Methodology, Data curation, Writing – review & editing. **Marcel van de Wouw:** Methodology, Data curation, Writing – review & editing.

## Declaration of Competing Interest

The authors declare that they have no known competing financial interests or personal relationships that could have appeared to influence the work reported in this paper.

## Data Availability

Pregnancy during the COVID-19 Pandemic Study (Original data) (Open Science Framework).
